# Environmental and Individual Predictors of Healthy Dietary Behaviors in a Sample of Middle Aged Hispanic and Caucasian Women

**DOI:** 10.3390/ijerph15102277

**Published:** 2018-10-17

**Authors:** Deborah J. Bowen, Jennifer M. Jabson, Wendy E. Barrington, Alyson J. Littman, Donald L. Patrick, Anne Vernez Moudon, Denise Albano, Shirley A. A. Beresford

**Affiliations:** 1 Bioethics and Humanities, School of Medicine, University of Washington, 1107 NE 45th Street #305, Seattle, WA 98105, USA; 2 Department of Public Health, University of Tennessee, Knoxville, TN 37996, USA; jjabson@utk.edu; 3 Psychosocial & Community Health, School of Nursing, University of Washington, Seattle, WA 98195, USA; wendybar@uw.edu; 4 VA Puget Sound Health Care System, Seattle Epidemiologic Research and Information Center, Seattle, WA 87185, USA; alyson@uw.edu; 5 VA Puget Sound Health Care System, Center of Innovation for Veteran-Centered and Value-Driven Care, Seattle, WA 98195, USA; 6 Epidemiology, School of Public Health, University of Washington, Seattle, WA 98195, USA; dalbano@fredhutch.org (D.A.); beresfrd@uw.edu (S.A.A.B.); 7 Health Services, School of Public Health, University of Washington, Seattle, WA 98195, USA; donald@uw.edu; 8 Urban Design & Planning, Architecture, Landscape Architecture, University of Washington, Seattle, WA 98195, USA; moudon@uw.edu

**Keywords:** dietary intake, socioeconomic status, women, obesity

## Abstract

The objective of this effort is to gather data to tailor interventions appropriately. Greater understanding of the correlates of socioeconomic status and obesogenic dietary behaviors was the focus of this manuscript. Using multistage sampling, women with varied education levels completed a baseline assessment in a longitudinal study of women aged 30 to 50 years. This study was conducted in low-SES areas of South King County, Washington State. This study included 530 Caucasian and 510 Hispanic women. Fruit and vegetable consumption was positively associated and soft drink consumption inversely associated with the level of education in Caucasian women. In contrast, percentage calories from fat was positively associated with the level of education in Hispanic women. In Hispanic women, level of education interacted significantly with food security in relation to percentage calories from fat, and with eating norms in relation to soft drink consumption. Neighborhood presence of ethnic food stores was associated with outcomes for Hispanic women, but for Caucasians, presence of fast food restaurants was important. Education was consistently associated with two of the three obesogenic dietary behaviors studied among Caucasian women. Education played a moderating role in the associations of food security and eating norms, independent of area level food availability, in two of three obesogenic dietary behaviors studied. However, these patterns differed for Hispanic women, indicating the need for more research into important variables to support change in Hispanic women. Women of differing ethnic groups did not respond similarly to environmental conditions and policy-relevant surroundings. These data have meaning for considering urban policy that impacts obesity levels in the population.

## 1. Introduction

Obesity occurs disproportionately in women of lower socioeconomic status (SES), especially in industrialized countries [1]. The SES–obesity relationship has been demonstrated across the life-course, beginning as early as adolescence with strong tracking from childhood into adulthood [2,3,4]. Additionally, women often play an important role in shaping the obesity-related (i.e., “obesogenic”) behaviors of family members [5]. Therefore, gaining a full understanding of the SES–obesity relationship during the middle years for women, and the role of these obesogenic behaviors in that relationship are important for identifying clues for prevention.

Dietary behaviors associated with both obesity and excess energy intake include: low consumption of fruits and vegetables [6], high consumption of fat [7], and high consumption of sugar-sweetened beverages. Low income and low educational attainment has been associated with low consumption of fruits and vegetables and high intake of fast food meals, soft-drinks, and foods high in sugar and fat [8]. Exploring the roles of psychosocial and environmental variables together with SES may help explain these relationships and could provide mechanisms for possible intervention.

### 1.1. Psychosocial Factors and Dietary Behaviors

Psychosocial factors are relevant individual-level predictors of human behavior because they are characteristics of an individual that are influenced by the social and physical environments and may help explain relationships between SES and behavior [9]. However, models including more traditional psychosocial variables (i.e., knowledge, attitudes, beliefs) have accounted for less than a third of the variance of dietary behaviors [10], calling for further expansion of psychosocial models predicting dietary behavior [11,12]. Potential psychosocial predictors of dietary behavior include perceived access to quality food, personal and perceived social norms, and perceived behavioral control [11,12]. Evaluation of whether relationships between psychosocial factors and dietary behaviors differ for low-SES and minority women is also needed [13].

The price of food has been found to be one significant barrier to accessing healthy food [8,11]. Perceptions surrounding the availability of healthy food outlets have also been shown to predict fruit and vegetable as well as fast food consumption [14,15]. Perceptions of availability might not always line up with objective measures of availability of healthy foods, as the elements of accessibility include availability (e.g., presence in neighborhood), affordability, accessibility (e.g., store hours), acceptability, and accommodation [16]. The relationship of perceived or actual availability of healthy foods with socioeconomic status is not clear [5,17] and may involve additional SES-related factors such as car-ownership [17].

Perceived personal and social norms around healthy eating and weight has been associated with dietary behaviors [1,11] and obesity [18]. Personal norms pertain to what an individual decides is appropriate behavior in a given situation whereas social norms are influences which arise from the social environment itself such as culture or other venues of social interaction [19]. A few studies suggest the relationship between eating and weight-related norms may differ by both ethnicity and SES (e.g., Reference [18]), thus, there is an argument for analyzing these separately. Further research is needed to confirm these findings, especially among non-White women.

### 1.2. Environmental Factors and Dietary Behaviors

The evidence for an association between greater access to healthy food outlets and healthier diets and lower levels of obesity is mixed [20,21,22,23]. Availability of healthy food outlets has been demonstrated to be lower among many low-SES and minority neighborhoods [24]. Yet, there remains insufficient evidence concerning the interactions of neighborhood availability of food outlets with socioeconomic status of individuals [25]. We hypothesize that these interactions shape obesogenic behaviors and differ by SES. We defined several measures of the SES-relevant food environment by reviewing the literature for possible SES-relevant factors. In the present paper, these variables will be tested in interaction models in relationship to obesogenic behaviors.

With respect to environmental variables, Ding and colleagues recently published a paper that evaluated the association of several individual and neighborhood activity-related variables with multiple physical activity behaviors [26]. They systematically tested interactions of psychosocial variables (e.g., self-efficacy, barriers to exercise) with neighborhood characteristics (e.g., presence of exercise facilities) in relationship to physical activity. These analyses allowed the investigators to identify overall patterns in the relationships of interacting environmental and individual variables as related to physical activity. We used Ding et al.’s [26] approach as a model for our analyses here, to identify patterns of interaction that will improve our understanding of SES and obesogenic behaviors. This in turn may guide the development of intervention strategies to reduce obesity across SES status.

The purpose of this paper is to explore the association of multiple individual and environmental variables and their interaction with selected psychosocial variables on obesogenic dietary behaviors in a sample of women recruited for SES diversity. First, we identified the relationship between socioeconomic status and these individual-level variables. We considered models, using an approach modified from Ding et al., 2012 [26] to identify patterns of interactions from our list of possible psychosocial and neighborhood moderators.

## 2. Materials and Methods

### 2.1. Study Design and Recruitment

The Socioeconomic Status and Obesity Study (The SESO Study) enrolled Hispanic and Caucasian women in primarily low-SES areas of South King County, Washington during 2010–2012 to evaluate associations between SES and obesity. The study was designed to test three specific mediating pathways between SES and obesity including: (1) access to material resources in the neighborhood; (2) the psychosocial context; and (3) the stress process [27,28]. These cross-sectional analyses use baseline data from this longitudinal study. All subjects gave their informed consent for inclusion before they participated in the study. The study was conducted in accordance with the Declaration of Helsinki, and the protocol was approved by the Ethics Committee of the Fred Hutchinson Cancer Research Center (9511).

Women were recruited into the SESO study using population-based sampling methods where the first stage involved selecting U.S. Census block-groups within Seattle with a high representation of Hispanics, low education and low income as determined by the 2000 U.S. Census (*n* = 143 block-groups). These methods are detailed elsewhere [29]. In brief, groups of houses were randomly selected within these selected block-groups and were approached by female study interviewers to determine the residency of an eligible woman within the selected households. Eligibility criteria included being a woman, aged 30–50 years, who spoke English or Spanish and who was not planning to move out-of-area in the next 3 years (to facilitate longitudinal follow-up). We selected this age group because it is a time of steady weight gain for adult women [4]. Potential study participants were selected at the screening stage to oversample women with fewer years of education among the Caucasian group. At baseline, 1040 (530 Hispanic, 510 Caucasian) women provided both screening and survey data. Each cohort was analyzed separately.

### 2.2. Procedures

We recruited all Hispanic women using these strategies. Eligible Caucasian women whose highest education included completing high school or earning a diploma equivalent (GED), 4% with some college through post-bachelor course or training, and 50% with a Master’s or doctoral or professional degree were randomly sampled to join the study, due to the larger numbers of eligible Caucasian women. Supplementary Table S1 shows missing data for all key variables.

### 2.3. Measures

Socioeconomic status. For this analysis, we defined socioeconomic status at the individual-level by years of education in five categories for Caucasian women (some high school/diploma/GED, vocational/training after high school, some college/Associates degree, college graduate, professional school after college, and graduate degree) and six categories for Hispanic women (did not go to school or grade school (1–4 years), grade school (5–8 years), some high school, high school diploma or GED, vocational school/some college/Associates degree, college graduate/Baccalaureate degree or higher). These differences in coding were due to the large differences in distribution between Hispanic and Caucasian women in this sample. Education was treated as a continuous variable.

Unaffordability of food. Perceptions of fruit and vegetable affordability, and the affordability of food in general, were measured with a modified version of Disball and colleagues’ [30] instrument. Six individual items were answered by participants, including items such as, “I think vegetables are affordable to me where I buy most of my food”, and “Lack of money prevents me from eating healthfully”. Responses were scaled from ‘strongly agree’ (1) to ‘strongly disagree’ (4). Factor analysis was performed as described above, and two factors were identified. Responses were averaged to form scales for the variables identified in the principal components. The first scale was labeled ‘food unaffordable’, and included four items such as “buying vegetables is difficult on my budget” and “buying fruit is difficult on my budget”. The Cronbach’s alpha was 0.89 for the food unaffordable scale. Larger values indicated that food was perceived as unaffordable. The second scale was labeled ‘fruits and vegetables affordable’, and included two items: “I think vegetables are affordable to me where I buy most of my food”, and “I think fruits are affordable where I buy most of my food”. The Cronbach’s alpha was 0.84 for the ‘fruits and vegetables affordable’ scale. Larger values indicated that fruits and vegetables were perceived as affordable.

Perceived food insecurity. Food insecurity was measured and scored according to the United States Department of Agriculture food security adult module [31]. Participants answered six questions regarding their food security, including such items as, “In the past 12 months, we worried whether our food would run out before we got money to buy more” and “In the last 12 months, we couldn’t afford to eat balanced meals”. Affirmative responses to the six items were summed (range 0–6) and then coded according to the USDA’s guidelines for food security. Participants with zero or one affirmative responses were labeled “food secure”. Participants with two to four affirmative responses were labeled “food insecure without hunger”, and those with five to six affirmative responses were labeled “food insecure with hunger”. Larger values indicated greater food insecurity.

Perceived availability of grocery stores/areas for exercise. Availability of grocery markets and areas designed for exercise were each assessed with one item modified from the Neighborhood Environment Walkability Scale (NEWS) [32]. Participants answered seven questions regarding neighborhood characteristics, scaled from ‘strongly agree’ (1) to ‘strongly disagree’ (4), where higher scores indicated more favorable neighborhood characteristics. Principal component analysis, with varimax rotation, was calculated on these seven items. One factor was retained; individual loadings were equal to or greater than 0.60 on a single factor and less than 0.40 on any other factor. Responses were averaged for the variables identified in the principal components. The Cronbach’s alpha was 0.71 for the three items in the newly formed scale. Items included questions such as, “there are many grocery stores and supermarkets in my neighborhood”, “there are many places to exercise in my neighborhood such as gyms, parks, and fitness trails”, and “There are many places to go within easy walking distance to my home”. Higher values indicated more access to grocery markets and areas designed for exercise.

Eating Norms. Measures of eating norms were created based on the work of Christakis, Fowler and colleagues [33] that investigated the spread of obesity in social networks. Two questions asked participants how many of their five closest friends, and second, how many of their five closest family members, “…eat a healthy diet?”. Responses could range from 0–5. These two questions were averaged to provide a single social norm score for eating score where larger values indicated having more friends/family that eat a healthy diet.

Weight-related Norms. Weight norms were assessed based on published work by Christakis, Fowler and colleagues [33]. Two questions were also used to assess weight norms. Participants were asked first, how many of their five closest friends, and second, how many of their five closest family members, “…were overweight?”. Responses could range from 0–5 and responses were averaged to form a single social norm for weight-related score where larger values indicating having more friends/family who are overweight.

Food management. Food management was measured according to Dickin and colleague’s [33] six-item food management measure. Questions included items such as, “How often do you plan meals ahead of time”, “How often do you run out of food before the end of the month”, and “How often do you shop with a grocery list?”. Responses were on a Likert five-point scale from “Do not do” (1) to “Almost always” (5). The six items were summed and then converted to a 100-point scale for ease of interpretation. Higher scores indicated better food management behaviors.

Objective environment: availability of neighborhood food outlets. Neighborhood was defined by geocoding the participants address and forming a concentric circle with a radius of 0.5 miles. The density of food outlets were enumerated within this buffer using geospatial data from University of Washington’s Urban Form Laboratory. Geocoded addresses of food establishments were based on food permit data from the Seattle King County Health Department and were verified via cross-checking with online business directories. Categorizations of food outlets extracted included: food stores (including broad-selection groceries and specialty produce markets), ethnic grocery stores, and fast food or “quick-serve” restaurants (e.g., where food is already prepared and packaged) [17,34].

### 2.4. Dependent Variables

We identified three obesogenic behaviors as dependent measures for these analyses. They included: (1) daily fruit and vegetable consumption [35], a single item measure used in previous research to quantify the number of fruit and vegetable servings consumed daily (“How many servings of fruits and vegetables do you eat each day?“); (2) soft drink consumption [36] using a single item measure used in previous research to quantify the frequency with which soft drinks are consumed (how often do you drink soft drinks or soda pop (*regular or diet*)?); and (3) % energy consumed as fat from the 24-h dietary recalls [37] as a nutrient density estimate. The unannounced 24-h recall(s) were conducted using the Nutrition Assessment Shared Resource, which uses a computer-assisted telephone interviewing approach, with multiple passes through the 24-h period to obtain overview, detail and checks of all foods consumed. A booklet to assist in the process was mailed out in advance of the unannounced call(s) by about 1 week. We used the University of Minnesota Nutrition Data System for Research database as a reference. For all dependent variables, larger values indicated higher levels of consumption. All of these measures have been used with reasonable performance in studies previously and all have been linked to obesity in other research.

### 2.5. Demographic Variables and Covariates

Participants provided demographic information including ethnicity (Hispanic/non-Hispanic), highest level of education completed, age at baseline data collection, level of acculturation, marital status, highest level of education achieved, total family income, and employment status.

### 2.6. Statistical Analyses

Descriptive and summary statistics were calculated for participants’ demographic characteristics. Descriptive statistics were calculated on the three dependent variables including percentage calories consumed from fat, daily fruit and vegetable consumption, and soft drink consumption, as well as all associated variables, by education and ethnicity.

Multi-level linear regression models with a random block group effect were used to estimate associations between SES (i.e., education) and dietary behaviors (i.e., daily fruit and vegetable servings, percentage calories from fat, and weekly soft drink intake) to account for participant clustering in neighborhoods [26]. Each model was fitted with one SES-related psychosocial variable, one neighborhood environment food outlet variable, and two interaction terms; one interaction term that included education and a neighborhood food-related variable and one interaction term that included psychosocial and a neighborhood food-related variable. Model covariates included age for all models and acculturation for Hispanic-only models. Two dependent variables (fruit and vegetable consumption and percent calories from fat) were non-normally distributed. A ladder of powers test was conducted to identify square root transformation as the optimal transformation to convert non-normally distributed variables to a normal distribution. An alpha level of 0.05 was used to identify significance. All statistical analyses were performed using Stata SE, version 11.0 (StataCorp, College Station, TX, USA). Supplemental Table S2a–c contains regressions with no significant interactions and Supplemental Table S3a–c focuses on variables with significant interactions in full.

## 3. Results

Pregnant women (*n* = 6 Hispanic and *n* = 10 Caucasian) and women with missing data for the variables of interest were excluded from the sample for analyses (Supplemental Table S1). The final analytic sample included *n* = 1002 (509 Hispanic and 493 Caucasian). We calculated a response rate of 46% using methods articulated from AAPOR [38].

Table 1 presents demographic data for the women in the analytic dataset included in the paper. The distribution of educational levels was different for the two different cohorts, but because of the sampling design there was a sizable sample of women with high school or less education (low-SES) in each cohort. As seen in Table 1, there was appreciable variability in age, family structure, and income within each study cohort.

Table 2 presents the relationships between educational level and the three dependent variables for Caucasian and Hispanic women. As seen in the upper part of this table, several dependent variables differed by educational level of the woman in both cohorts. For White women, two of three dependent variables differed by education level, such that less educated women ate fewer fruits and vegetables daily (*p* < 0.001) and consumed more soft drinks (*p* < 0.001). The same pattern of behaviors did not hold true for Hispanic women. Hispanic women reported higher levels of fat consumption in the higher three education categories, with significant differences across education level (*p* = 0.01).

Table 2 also contains the psychosocial association variables by educational level for both cohorts. As seen in this table, many of these variables differed by education level in both cohorts. Food insecurity was lowest in Caucasian women of higher education levels, (*p* < 0.01). Food management, fruit/vegetable affordability, and food affordability judgments differed across education level for Caucasians (*p*’s < 0.001). In all cases, higher SES Caucasians reported less obesogenic psychosocial association variables. Finally, weight and eating norms differed across education levels for Caucasian women (*p*’s < 0.001), with higher weight norms for lower education levels and lower eating norms for lower education levels. For Hispanic women, food unaffordability, food management, and grocery/activity access scores differed across education levels (*p*’s < 0.001) and so did social norms for eating (*p*’s < 0.001), in the same direction as for Caucasian women.

Table 3, Table 4 and Table 5 presents the results of final models testing the interaction terms. Supplemental Tables S2a–c and S3a–c present the results of models with main effects only, to aid in interpretation of the final models. Each model table presents similarly structured models for one of the obesogenic dietary behaviors as dependent variables, adjusted for age. For each table, the psychosocial variables are listed down the left hand side, and the neighborhood-level variables are listed in three columns. For each final regression model, the main effects of education, the psychosocial variable, and the neighborhood-level variable are listed, followed by the interaction terms of education by the psychosocial and the neighborhood-level variable, for a total of five regression coefficients displayed per model. All variables are referred to by initials for simplicity.

Table 3 presents the group of model findings for fruit and vegetable consumption as the dependent variable. Education was significantly and directly associated with outcomes in several of the models for Caucasians, but there were no significant interactions with education. For Hispanic women, significant interactions of education with ethnic food store, were found in relation to fruit and vegetable consumption in the models that included fruit and vegetable affordability (inversely), grocery store access (inversely), and both weight and eating norms (both inversely). Of all the psychosocial variables, only eating norms were related to fruit and vegetable consumption for Hispanic women in one the three models. The presence of ethnic food stores in the surrounding community was related to fruit and vegetable consumption for all of the seven models in Hispanic women. Most interactions were significantly related to fruit and vegetable consumption with a few exceptions.

Table 4 presents the group of model findings for soft drink consumption. For Caucasian women, there were no significant interactions with education. For Hispanic women, only the interaction between education and eating norms in relation to soft drink consumption in the model that included fast food restaurants was significant. Education was significantly and inversely related to soft drink consumption for Caucasian women in four of the models. Food insecurity scale (inversely), food unaffordable scale (inversely), and eating norms (inversely) were all related to soft drink consumption for Caucasian women. For Hispanic women, an inverse association between education and soft-drink consumption was suggested, albeit not statistically significant, in models including food insecurity and weight norms. Furthermore, no psychosocial variables were independently related to soft-drink consumption for Hispanic women. The interaction of eating norms and education was also inversely related to soft drink consumption for Hispanic women.

Table 5 presents the group of model findings for percentage calories from fat. For Caucasian women, there were no significant interactions with education. However, there was a direct and significant effect of presence of fast food outlets in the environment on percentage calories from fat for Caucasian women. For Hispanic women, the direct effects of food security in relation to percentage calories from fat were significant in all models, as was the interaction between food security and education. The effect of eating norms and food management were inverse and significant for all models in Hispanic women.

## 4. Discussion

We set out to test interactions between environmental and individual-level variables relevant to obesity in Caucasian and Hispanic women. Education is clearly related to fruit and vegetable consumption, mostly for Caucasian women. For Caucasian women, the effect of education was in the hypothesized direction across the five categories, showing an inverse relationship between education and healthy eating. For Hispanic women, the relationship was more complex and not consistent across dependent variables. This finding has been noted in the literature, as Hispanic women’s education levels do not relate consistently and linearly to their BMI [29]. Further exploration of the correlates of weight in Hispanic women is clearly needed.

The modeling of psychosocial variables, neighborhood-level variables, and interactions helped us to identify patterns of findings that might lead to modifiable relationships of elements of behavior and environment with obesogenic dietary behaviors. Again, the patterns differed by ethnicity. For Caucasian women, eating norms and food management levels, and to a lesser extent, food insecurity and food affordability perceptions were all related to obesogenic behaviors. For Hispanic women, eating norms was the only consistent psychosocial variable related to obesogenic behaviors. One possible explanation for these patterns is that the levels of some of these variables were different between the two cohorts. In general, Hispanic women had different food insecurity ratings and lower food management and affordability ratings, possibly pushing Hispanic women into a part of the distribution for these variables that is simply underserved and therefore, does not vary enough to see differences.

Weak or no associations were observed between many neighborhood-level variables and obesogenic dietary variables. There were two consistent exceptions to this general finding: The association between the presence of ethnic food stores and fruit and vegetable consumption for Hispanic women and the positive association between presence of fast food restaurants and both percentage calories from fat and soft drink consumption in Caucasians. Regarding the finding in Hispanic women, this could be of critical importance for future studies that attempt to increase fruit and vegetable consumption among Hispanic women, who might need access to fruits and vegetables that they recognize and feel comfortable with. There is some evidence for the presence of food outlet subtypes as differently related to different groups of adults and children’s behaviors [39] and so this needs confirmation in other samples. The presence of fast food restaurants was associated with percentage calories from fat and soft drink consumption in models for Caucasian but not for Hispanic women, which given that both cohorts were exposed to similar neighborhood environments, suggests cultural differences in the health impact of these restaurants. Given the mixed results in studies of neighborhood surroundings and obesity [40], this line of research needs more investigation.

This study has several design qualities that limit the generalizability of the findings. First, the Hispanic and Caucasian women were sampled from the same neighborhoods, and so the interpretation of the findings is limited to women who reside in more integrated neighborhoods. All neighborhoods in Seattle are not so integrated, and the extent of integration has been related to other health and risk outcomes in previous research [41,42]. Future research could sample differently and therefore include racial isolation as a variable in analyses. We certainly lost potential participants to data collection due to study burden, an inevitable part of research, but one that tends to work more heavily with persons of lower SES from participation. We mindfully sampled from a variety of socioeconomic strata, but we still lost participants, likely not at random. So, this study group may not completely represent the geographic areas from which it was sampled. In addition, we did not use a Bonferroni or other adjustment for multiple testing in our statistical analysis. Instead, we simply did not remark on findings that were related by less than a 0.05 value and looked for consistency of patterns and interpreted those rather than individual statistical significance tests.

This study does include strengths that enhance the value of its findings. It is unusual in that it was explicitly designed to include women of all SES levels, to enable the evaluation of education on obesity related variables. Therefore, we were able to analyze these data across educational levels with some samples in each important cell, although the sizes did get small in some of the lower SES levels for non-Hispanic white women. It is a sample selected based on household location and not completely through convenience or volunteering, thus increasing the nonbiased selection of participants. Finally, it includes cohorts of two different ethnic groups, thereby increasing our ability to understand the role of SES in women from many diverse backgrounds.

## 5. Conclusions

In summary, education is consistently associated with two of the three obesogenic dietary behaviors studied among Caucasian women and for these women, education plays a moderating role in the associations of food security and eating norms, independent of area level food availability, in two of three obesogenic dietary behaviors studied. For Hispanic women, there were a few individual variables that related to obesogenic dietary behaviors, but the neighborhood presence of ethnic food stores consistently related to these behaviors and could be a direction for future investigation and public health practice. Considering these findings when creating or modifying policies related to area-level obesogenic policies, laws, and regulations makes sense in the context of urban planning.

## Figures and Tables

**Table 1:** Individual-level study characteristics, by ethnicity. Table may not sum to 100% due to missing data.

Characteristics	Caucasian		Hispanic	
	%	(*n*)	%	(*n*)
Overall	49.40	493	50.60	509
Age				
30–<35	26.90	131	30.72	149
35–<40	26.28	128	32.58	158
40–<45	22.18	108	21.24	103
45–<51	24.64	120	15.46	75
Accultruation				
Spanish	0.20	1	84.00	420
English	99.80	488	16.00	80
Marital Status				
Never married/divorced/separated/widowed	28.98	142	22.42	113
Married/living with partner	71.02	348	77.58	391
Education Level				
High school graduate or lower	20.89	103		
Didn’t go to school/1–4 years			11.98	61
Grade School/5–8 years			22.79	116
Some High School			16.50	84
High School Graduate/GED			25.15	128
Some college or Associate Degree	29.82	147	13.56	69
College graduate or higher			10.02	51
College graduate or bacculaureate degree	23.73	117		
Professional/Graduate Degree	20.08	99		
Total Family Income				
<$30K	21.58	101	62.97	250
$30K–<$50K	14.53	68	22.17	88
$50k–<$75K	21.58	101	9.32	37
$75K–<$100K	18.80	88	3.53	14
≥$100K	23.50	110	2.02	8
Employment				
Other	33.60	165	52.08	263
Employed (full-time, part-time, self-employed)	66.40	326	47.92	242

**Table 2:** Obesogenic behaviors and psychosocial factors by education level and ethnicity. Table may not sum to 100% due to missing data.

	Caucasian							Hispanic						
	(*n* = 493)							(*n* = 509)						
		Some High School (9–11 Years) & Diploma or GED	Vocational or Training after HS/Some College/Associates Degree	College Grad/Bachelors Degree	College/Prof. School after College	Graduate Degree		Didn’t Go to School, Grade School (1–4 Years)	Grade School (5–8 Years)	Some HS (9–11 years)	HS Diploma or GED	Voc/Training School, Some College/Associates Degree	College Graduate or Baccalaureate Degree or Higher	
		(*n* = 103)	(*n* = 147)	(*n* = 117)	(*n* = 27)	(*n* = 99)		(*n* = 61)	(*n* = 116)	(*n* = 84)	(*n* = 128)	(*n* = 69)	(*n* = 51)	
**Dependent Variables**		mean (sd)					*p*	mean (sd)						*p*
	*n*													
Number of daily fruit/vegetable consumed	490/495	2.66 (1.68)	3.32 (1.76)	3.60 (1.79)	4.44 (2.26)	4.31 (1.66)	<0.001	2.46 (1.79)	2.75 (1.89)	2.83 (1.69)	2.57 (1.54)	2.79 (1.49)	2.84 (1.87)	0.42
% calories fat	421/387	34.64 (10.51)	34.54 (10.39)	34.65 (9.38)	34.93 (7.44)	34.00 (8.97)	0.98	29.95 (9.22)	27.10 (9.21)	27.69 (9.19)	30.88 (8.58)	30.96 (9.94)	31.68 (7.69)	0.01
Number of daily soft drinks consumed	492/495	2.63 (1.74)	2.07 (1.66)	1.56 (1.48)	1.52 (1.40)	1.59 (1.49)	<0.001	2.18 (1.35)	2.12 (1.45)	2.41 (1.44)	2.30 (1.38)	2.25 (1.41)	1.75 (1.42)	0.13
**Psychosocial Variables**														
Food Insecurity	483/465	2.09 (2.25)	1.18 (1.87)	0.30 (1.00)	0.48 (1.22)	0.21 (0.95)	<0.001	2.05 (1.75)	1.72 (1.72)	1.65 (1.59)	1.60 (1.73)	1.64 (2.07)	1.38 (2.14)	0.55
Fruits/Vegetables Affordable	484/492	2.21 (0.73)	1.99 (0.75)	1.74 (0.67)	1.70 (0.74)	1.57 (0.74)	<0.001	1.99 (.84)	2.19 (0.72)	2.22 (0.78)	2.17 (0.78)	2.10 (0.90)	2.09 (.87)	0.59
Food Unaffordable	473/475	2.72 (0.77)	2.98 (0.78)	3.36 (0.66)	3.36 (0.55)	3.51 0 (.54)	<0.001	2.43 (.84)	2.64 (0.75)	2.58 (0.64)	2.81 (0.67)	2.89 (0.80)	2.88 (.80)	0.001
Food Management	431/453	56.16 (16.39)	59.56 (16.64)	64.54 (10.68)	66.32 (15.14)	64.26 (13.07)	0.007	42.69 (22.35)	47.96 (19.65)	46.96 (17.99)	47.58 (19.55)	59.72 (17.00)	55.87 (18.81)	<0.001
Grocery/Exercise Access	484/491	2.00 (0.64)	2.01 (0.69)	3.12 (1.19)	2.17 (.75)	1.82 (0.64)	0.05	1.62 (0.56)	1.90 (0.67)	2.02 (0.60)	1.91 (0.62)	2.04 (0.66)	1.89 (.60)	0.002
Weight Norms	484/475	2.55 (1.10)	2.18 (1.26)	1.85 (1.10)	1.67 (0.98)	1.57 (1.00)	<0.001	2.01 (1.39)	2.05 (1.34)	2.21 (1.21)	2.18 (1.39)	2.49 (1.34)	2.12 (1.09)	0.33
Eating Norms	482/467	2.13 (1.11)	2.67 (1.32)	3.12 (1.19)	3.69 (1.08)	3.60 (1.09)	<0.001	1.47 (1.62)	1.41 (1.41)	1.51 (1.36)	1.37 (1.33)	2.26 (1.55)	2.48 (1.32)	<0.001

**Table 3:** Associations of psychosocial and environmental variables with fruit and vegetable consumption, by ethnicity, adjusted for age.

	Caucasian						Hispanic					
	(*n* = 493)						(*n* = 509)					
	Area−Level Variables											
	Supermarkert/Grocery Stores (SG)		Ethnic Food Stores (EF)		Fast Food Restaurants (FF)		Supermarkert/Grocery Stores (SG)		Ethnic Food Stores (EF)		Fast Food Restaurants (FF)	
Psychosocial Variables		*b*						*b*				
Food Insecurity (FI) (*n* = 480/420)	FI	0.01	FI	0.01	FI	0.01	FI	0.01	FI	0.007	FI	0.007
Education (E)	E	0.12 **	E	0.11 **	E	0.13 **	E	0.003 *	E	0.04	E	0.03
	SG	0.11	EF	0.07	FF	0.09	SG	−0.19	EF	0.28 *	FF	0.09
	FI x E	−0.02	FI x E	−0.001	FI x E	−0.02	FI x E	−0.01	FI x E	−0.01	FI x E	−0.01
	SG x E	−0.02	EF x E	−0.02	FF x E	−0.03	SG x E	0.05	EF x E	0.05	FF x E	−0.02
Fruit/Vegetable Affordable (FA) (*n* = 481/449)	FA	−0.09	FA	−0.09	FA	−0.09	FA	0.03	FA	0.03	FA	0.04
	E	0.10 *	E	0.07	E	0.10 *	E	0.03	E	0.07	E	0.06
	SG	0.10	EF	0.04	FF	0.07	SG	−0.12	EF	0.3 *	FF	0.11
	FA x E	0.01	FA x E	0.01	FA x E	0.01	FA x E	−0.02	FA x E	−0.02	FA x E	−0.02
	SG x E	−0.02	EF x E	0.01	FF x E	−0.02	SG x E	0.03	EF x E	−0.06 *	FF x E	−0.04
Food Management (FM) (*n* = 476/409)	FM	0.01 **	FM	0.01 **	FM	0.01 **	FM	0.006	FM	0.006	FM	0.005
	E	0.23 *	E	0.21 *	E	0.23 *	E	−0.03	E	0.001	E	−0.01
	SG	0.12	EF	0.07	FF	0.06	SG	−0.14	EF	0.30 *	FF	0.16
	FM x E	−0.002	FM x E	−0.002	FM x E	−0.002	FM x E	0.0003	FM x E	0.0004	FM x E	0.0005
	SG x E	−0.02	EF x E	0.01	FF x E	−0.01	SG x E	0.02	EF x E	−0.03	FF x E	−0.05
Food Unaffordable (FU) (*n* = 476/435)	FU	0.12	FU	0.13 *	FU	0.11	FU	−0.003	FU	0.01	FU	0.01
	E	0.15	E	0.15	E	0.14	E	−0.1	E	−0.05	E	−0.07
	SG	0.10	EF	0.06	FF	0.07	SG	−0.14	EF	0.26 *	FF	0.07
	FU x E	−0.01	FU x E	−0.02	FU x E	−0.009	FU x E	0.03	FU x E	0.03	FU x E	0.03
	SG x E	−0.02	EF x E	0.002	FF x E	−0.02	SG x E	0.03	EF x E	−0.05	FF x E	−0.02
Grocery/Exercise Access (GE) (*n* = 481/443)	GE	−0.03	GE	−0.03	GE	−0.02	GE	−0.16	GE	−0.13	GE	−0.14
	E	0.13 *	E	0.12 *	E	0.15 *	E	−0.04	E	0.02	E	−0.001
	SG	0.10	EF	0.08	FF	0.12	SG	−0.16	EF	0.31 *	FF	0.11
	GE x E	−0.006	GE x E	−0.004	GE x E	−0.01	GE x E	0.02	GE x E	0.01	GE x E	0.02
	SG x E	−0.02	EF x E	−0.004	FF x E	−0.04	SG x E	0.03	EF x E	−0.07 *	FF x E	−0.04
Weight Norms (WN) (*n* = 481/432)	WN	0.003	WN	0.001	WN	0.004	WN	−0.01	WN	−0.01	WN	−0.01
	E	0.13 **	E	0.12 *	E	0.14 **	E	−0.004	E	0.04	E	0.02
	SG	0.07	EF	0.04	FF	0.07	SG	−0.13	EF	0.31 *	FF	0.09
	WN x E	−0.007	WN x E	−0.006	WN x E	−0.008	WN x E	−0.003	WN x E	−0.004	WN x E	−0.003
	SG x E	−0.01	EF x E	−0.006	FF x E	−0.02	SG x E	0.03	EF x E	−0.06 *	FF x E	−0.03
Eating Norms (EN) (*n* = 480/485)	EN	0.12 *	EN	0.12 **	EN	0.12 *	EN	0.11	EN	0.12 *	EN	0.11 *
	E	0.16 **	E	0.16 **	E	0.17 **	E	−0.02	E	0.04	E	0.01
	SG	0.08	EF	0.05	FF	0.08	SG	−0.14	EF	0.34 *	FF	0.12
	EN x E	−0.02	EN x E	−0.02	EN x E	−0.02	EN x E	−0.005	EN x E	−0.009	EN x E	−0.007
	SG x E	−0.01	EF x E	0.006	FF x E	−0.02	SG x E	0.03	EF x E	−0.07 *	FF x E	−0.03

All models adjusted for age; Hispanic models adjusted for acculturation; * *p* ≤ 0.05; ** *p* ≤ 0.001.

**Table 4:** Associations of psychosocial and environmental variables with soft drink consumption, by ethnicity, adjuted for age.

	Caucasian						Hispanic					
	(*n* = 493)						(*n* = 509)					
	Area−Level Variables											
	Supermarkert/Grocery Stores (SG)		Ethnic Food Stores (EF)		Fast Food Restaurants (FF)		Supermarkert/Grocery Stores (SG)		Ethnic Food Stores (EF)		Fast Food Restaurants (FF)	
Psychosocial Variables	*b*						*b*					
Food Insecurity (FI) (*n* = 482/420)	FI	0.11 *	FI	0.12 **	FI	0.12 *	FI	−0.04	FI	−0.03	FI	−0.03
Education (E)	E	0.08 *	E	−0.07	E	−0.06	E	−0.06 *	E	−0.06	E	−0.06
	SG	−0.14	EF	−0.22	FF	0.01	SG	0.01	EF	−0.08	FF	0.02
	FI x E	−0.11	FI x E	−0.01	FI x E	−0.01	FI x E	0.009	FI x E	0.009	FS x E	0.007
	SG x E	0.07	EF x E	0.04	FF x E	0.02	SG x E	0.04	EF x E	0.02	FF x E	0.04
Fruit/Vegetable Affordable (*n* = 483/447)	FA	0.17	FA	0.20*	FA	0.18 *	FA	0.04	FA	0.04	FA	0.05
	E	−0.07	E	−0.05	E	−0.05	E	−0.04	E	−0.02	E	−0.04
	SG	−0.09	EF	−0.21	FF	0.06	SG	−0.09	EF	−0.10	FF	−0.09
	FA x E	−0.02	FA x E	−0.03	FA x E	−0.02	FA x E	−0.007	FA x E	−0.01	FA x E	−0.01
	SG x E	0.06	EF x E	0.05	FF x E	0.01	SG x E	0.06	EF x E	0.02	FF x E	0.06
Food Management (FM) (*n* = 430/413)	FM	−0.004	FM	−0.003	FM	−0.003	FM	−0.0001	FM	−0.0003	FM	−0.0001
	E	−0.04	E	−0.05	E	−0.04	E	−0.02	E	−0.01	E	−0.03
	SG	−0.06	EF	−0.18	FF	0.05	SG	−0.09	EF	0.003	FF	−0.04
	FM x E	−0.001	FM x E	−0.001	FM x E	−0.001	FM x E	−0.0006	FM x E	−0.0005	FM x E	−0.0006
	SG x E	0.03	EF x E	0.04	FF x E	0.02	SG x E	0.06	EF x E	0.01	FF x E	0.06
Food Unaffordable (FU) (*n* = 477/433)	FU	−0.29 *	FU	−0.32 **	FU	−0.30 **	FU	0.09	FU	0.10	FU	0.09
	E	−0.29 *	E	−0.31 *	E	−0.30 *	E	0.02	E	−0.03	E	−0.005
	SG	−0.07	EF	−0.21	FF	0.07	SG	−0.05	EF	−0.09	FF	−0.05
	FU x E	0.06	FU x E	0.07	FU x E	0.06	FU x E	−0.02	FU x E	−0.02	FU x E	−0.02
	SG x E	0.05	EF x E	0.04	FF x E	0.002	SG x E	0.05	EF x E	0.01	FF x E	0.05
Grocery/Exercise Access (GE) (*n* = 483/442)	GE	0.18	GE	0.19	GE	0.19	GE	0.10	GE	0.10	GE	0.11
	E	−0.04	E	−0.02	E	−0.01	E	0.003	E	0.03	E	0.005
	SG	−0.02	EF	−0.14	FF	0.09	SG	−0.09	EF	−0.05	FF	−0.07
	GE x E	−0.04	GE x E	−0.05	GE x E	−0.04	GE x E	−0.03	GE x E	−0.03	GE x E	−0.03
	SG x E	0.04	EF x E	0.03	FF x E	0.002	SG x E	0.06	EF x E	0.01	FF x E	0.05
Weight Norms (WN) (*n* = 483/430)	WN	0.07	WN	0.06	WN	0.07	WN	−0.05	WN	−0.05	WN	−0.04
	E	−0.11 *	E	−0.11 *	E	−0.09	E	−0.10 *	E	−0.10 *	E	−0.11 *
	SG	−0.02	EF	−0.15	FF	0.08	SG	−0.08	EF	−0.09	FF	−0.09
	WN x E	0.01	WN x E	0.01	WN x E	0.007	WN x E	0.02	WN x E	0.02	WN x E	0.02
	SG x E	0.05	EF x E	0.04	FF x E	0.009	SG x E	0.06	EF x E	0.03	FF x E	0.06
Eating Norms (EN) (*n* = 481/422)	EN	−0.20 **	EN	−0.21 **	EN	−0.20 **	EN	0.02	EN	0.02	EN	0.02
	E	−0.16 *	E	−0.17 *	E	−0.15 *	E	0.02	E	0.03	E	0.01
	SG	−0.04	EF	−0.06	FF	0.08	SG	−0.06	EF	−0.05	FF	−0.07
	EN x E	0.03	EN x E	0.03	EN x E	0.03	EN x E	−0.03	EN x E	−0.03	EN x E	−0.03 *
	SG x E	0.05	EF x E	0.04	FF x E	0.01	SG x E	0.06	EF x E	0.01	FF x E	0.04

All models adjusted for age; Hispanic models adjusted for acculturation; * *p* ≤ 0.05; ** *p* ≤ 0.001.

**Table 5:** Associations of psychosocial and environmental variables with percent calories from fat, by ethnicity, adjusted for age.

	Caucasian						Hispanic					
	(*n* = 493)						(*n* = 509)					
	Area−Level Variables											
	Supermarkert/Grocery Stores (SG)		Ethnic Food Stores (EF)		Fast Food Restaurants (FF)		Supermarkert/Grocery Stores (SG)		Ethnic Food Stores (EF)		Fast Food Restuarants (FF)	
Psychosocial Variables		*b*						*b*				
Food Inecurity (FI) (*n* = 411/333)	FI	0.25	FI	0.04	FI	0.10	FI	−10.32 *	FI	−10.31 *	FI	−10.31 *
Education E	E	0.09	E	−0.20	E	0.15	E	−0.06	E	0.12	E	−0.33
	SG	0.53	EF	20.08	FF	40.25	SG	0.83	EF	20.81	FF	−10.28
	FI x E	−0.25	FI x E	−0.19	FI x E	−0.21	FI x E	0.34 *	FI x E	0.34 *	FI x E	0.34
	SG x E	−0.8	EF x E	−0.16	FF x E	−0.88	SG x E	−0.62	EF x E	−0.75	FF x E	0.13
Fruit/Vegetable Affordable (FA) (*n* =413/356)	FA	−0.02	FA	−0.45	FA	−0.28	FA	0.60	FA	0.53	FA	0.51
	E	−0.21	E	−0.83	E	−0.25	E	0.94	E	0.93	E	0.54
	SG	10.12	EF	10.51	FF	40.67	SG	10.47	EF	20.75	FF	−0.96
	FA x E	0.21	FA x E	0.37	FA x E	0.31	FA x E	−0.14	FA x E	−0.09	FA x E	−0.08
	SG x E	−0.87	EF x E	−0.03	FF x E	−10.01	SG x E	−0.81	EF x E	−0.78	FF x E	0.01
Food Management (FM) (*n* = 372/332)	FM	−0.09	FM	−0.11	FM	−0.10	FM	−0.13 *	FM	−0.14 *	FM	−0.12 *
	E	−10.71	E	−20.25	E	−10.95	E	−0.81	E	−0.67	E	−0.94
	SG	10.27	EF	30.61	FF	40.09	SG	−0.13	EF	30.23	FF	−10.63
	FM x E	0.03	FM x E	0.04	FM x E	0.04	FM x E	0.03	FM x E	0.03	FM x E	0.03
	SG x E	−0.81	EF x E	−0.45	FF x E	−0.74	SG x E	−0.47	EF x E	−0.83	FF x E	0.17
Food Unaffordable (FU) (*n* = 412/346)	FU	0.70	FU	10.15	FU	0.68	FU	10.46	FU	10.34	FU	10.32
	E	10.46	E	10.66	E	10.52	E	20.02	E	20.15 *	E	10.8
	SG	0.83	EF	10.44	FF	40.29 *	SG	20.53	EF	30.91	FF	−0.08
	FU x E	−0.42	FU x E	−0.58	FU x E	−0.40	FU x E	−0.52	FU x E	−0.52	FU x E	−0.53
	SG x E	−0.83	EF x E	0.02	FF x E	−0.90	SG x E	−0.95	EF x E	−0.91	FF x E	−0.10
Grocery/Exercise Access (GE) (*n* = 413/352)	GE	−10.31	GE	10.92	GE	−10.25	GE	−0.63	GE	−0.72	GE	−0.89
	E	−0.21	E	−0.83	E	−0.26	E	0.14	E	−0.03	E	−0.34
	SG	0.25	EF	10.92	FF	40.40 *	SG	10.88	EF	20.05	FF	10.02
	GE x E	0.12	GE x E	0.31	GE x E	0.22	GE x E	0.26	GE x E	0.34	GE x E	0.37
	SG x E	−0.81	EF x E	−0.09	FF x E	−0.94	SG x E	−0.89	EF x E	−0.57	FF x E	−0.007
Weight Norms (WN) (*n* = 412/342)	WN	−0.71	WN	−0.72	WN	−0.65	WN	−0.56	WN	−0.54	WN	−0.76
	E	−0.71	E	−0.67	E	−0.17	E	0.23	E	0.34	E	−0.24
	SG	0.19	EF	20.11	FF	40.88 *	SG	0.89	EF	10.99	FF	−20.16
	WN x E	0.22	WN x E	0.25	WN x E	0.21	WN x E	0.19	WN x E	0.17	WN x E	0.23
	SG x E	−0.77	EF x E	−0.18	FF x E	−10.11	SG x E	−0.68	EF x E	−0.61	FF x E	0.28
Eating Norms (EN) (*n* = 411/335)	EN	−0.56	EN	−0.41	EN	0.5	EN	−10.72 *	EN	−10.72 *	EN	−10.74 *
	E	−0.34	E	−0.3	E	−0.05	E	0.62	E	0.72	E	0.42
	SG	0.56	EF	20.26	FF	50.20 *	SG	10.46	EF	20.27	FF	−10.55
	EN x E	0.16	EN x E	0.10	EN x E	0.14	EN x E	10.7	EN x E	0.16	EN x E	0.15
	SG x E	−0.88	EF x E	−0.37	FF x E	−10.20	SG x E	−0.8	EF x E	−0.67	FF x E	−0.03

All models adjusted for age; Hispanic models adjusted for acculturation; * *p* ≤ 0.05.

## Supplementary Materials

The following are available online at http://www.mdpi.com/1660-4601/15/10/2277/s1, Table S1: Frequency and percentage of missing dependent and independent variables, by ethnicity, Table S2a–c: For models without significant interaction terms, Associations of psychosocial and environmental variables with outcome variables, by ethnicity, adjusted for age. Table S3a–c: For models with significant interactions in main analyses, associations of psychosocial and environmental variables with outcome variables, by ethnicity, split by education, adjusted for age.
