# Supplementary material for: Environmental and Individual Predictors of Healthy Dietary Behaviors in a Sample of Middle Aged Hispanic and Caucasian Women
Source: Int J Environ Res Public Health. 2018 Oct 17;15(10):2277. doi: 10.3390/ijerph15102277 (PMC6210480; doi:10.3390/ijerph15102277)
Supplement: Supplementary file 1 [file ijerph-15-02277-s001.pdf]

Supplementary materials

Environmental and Individual Predictors of Healthy Dietary Behaviors in a Sample of Middle Aged Hispanic and Caucasian Women

Deborah J. Bowen, Jennifer M. Jabson, Wendy E. Barrington, Alyson J. Littman, Donald L. Patrick, Anne Vernez Moudon, Denise Albano and Shirley A. A. Beresford

Supplemental Table 1. Frequency and percentage of missing dependent and independent variables, by ethnicity

	Total (N = 1002)	CAUC (n = 493)	HISP (n = 509)
**Dependent Variables**		**n (%)**	
Fruit/vegetable consumption	17 (1.7)	3 (.06)	14 (2.7)
% calories fat	194 (19.4)	72 (14.6)	122 (24.0)
Soft drink consumption	15 (1.5)	1 (.02)	14 (2.8)
**Psychosocial Variables**			
Food Security	54 (5.4)	10 (2.0)	44 (8.6)
Fruits/Vegetables Affordable	26 (2.6)	9 (1.8)	17 (3.3)
Food Unaffordable	49 (4.9)	15 (3.0)	34 (6.7)
Food Management	118 (11.8)	62 (12.6)	56 (11.0)
Grocery/Exercise Access	27 (2.7)	9 (1.8)	18 (3.5)
Weight Norms	43 (4.3)	9 (1.8)	34 (6.7)
Eating Norms	53 (5.3)	11 (2.2)	42 (8.3)

Supplemental Table 2a. For models without significant interaction terms: Associations of psychosocial and environmental variables **with fruit and vegetable consumption**, by ethnicity, adjusted for age.

	Caucasian (n=493)						Hispanic (n=509)					
	**Area-Level Variables**											
	Supermarkert/Grocery Stores (SG)		Ethnic Food Stores (EF)		Fast Food Restaurants (FF)		Supermarkert/Grocery Stores (SG)		Ethnic Food Stores (EF)		Fast Food Restaurants (FF)	
**Psychosocial Variables**	*b*											
	FS	-0.02	FS	-0.02	FS	-0.02	FS	**-.04***	FS	**-.04***	FS	**-.04***
Food Security (FS)												
(n = 480/420)	E	**.11****	E	**.10****	E	**.11****	E	-0.005	E	-0.005	E	-0.005
Education (E)	SG	0.05	EF	0.07	FF	0.01	SG	-0.02	EF	0.1	FF	0.006
Fruit/Vegetable Affordable												
(FA) (n = 481/449)	FA	**-.06***	FA	**-.06***	FA	-0.06	FA	-0.04	FA	■	FA	-0.04
	E	**.11****	E	**.10****	E	**.11****	E	-0.00009	E	■	E	-0.000002
	SG	0.05	EF	0.060	FF	0.005	SG	-0.02	EF	■	FF	-0.02
Food Management (FM)												
(n = 476/409)	FM	**.008****	FM	**.008****	FM	**.008****	FM	**.007****	FM	**.007****	FM	**.007****
	E	**.10****	E	**.10****	E	**.10****	E	-0.01	E	-0.01	E	-0.01
	SG	0.06	EF	**.09***	FF	0.02	SG	-0.02	EF	0.09	FF	0.00009
Food Unaffordable (FU)												
(n = 476/435)	FU	**.10***	FU	**.09***	FU	**.09***	FU	**.10***	FU	**.10***	FU	**.10***
	E	**.10****	E	**.09****	E	**.10****	E	-0.005	E	-0.005	E	-0.004
	SG	0.05	EF	0.06	FF	0.009	SG	-0.03	EF	0.07	FF	-0.0007
Grocery/Exercise Access												
(GE) (n = 481/443)	GE	-0.04	GE	-0.05	GE	-0.05	GE	**-.09***	GE	■	GE	**-.08***
	E	**.11****	E	**.11****	E	**.11****	E	0.01	E	■	E	0.01
	SG	0.05	EF	0.06	FF	0.01	SG	-0.04	EF	■	FF	-0.01
Weight Norms (WN)												
(n = 481/432)	WN	-0.02	WN	-0.01	WN	-.02	WN	-0.02	WN	■	WN	-0.02
	E	**.11****	E	**.11****	E	**.11****	E	0.0001	E	■	E	0.0001
	SG	0.04	EF	0.06	FF	0.008	SG	-0.006	EF	■	FF	-0.009
Eating Norms (EN) (n =												
480/485)	EN	**.07****	EN	**.07****	EN	**.07****	EN	**.09****	EN	■	EN	**.09****
	E	**.10****	E	**.09****	E	**.10****	E	-0.02	E	■	E	-0.02
	SG	0.04	EF	0.06	FF	0.003	SG	-0.03	EF	■	FF	0.02

* all models adjusted for age; Hispanic models adjusted for acculturation; ** p ≤ .001; *p ≤ .05; x = no significant relationship identified in full models; ■ = significant interaction terms in main analyses and were not included in these supplemental analyses.

Supplemental Table 2b. For models without significant interaction terms: Associations of psychosocial and environmental variables **with soft drink consumption**, by ethnicity, adjusted for age.

	Caucasian (n=493)						Hispanic (n=509)					
	**Area-Level Variables**											
	Supermarkert/Grocery Stores (SG)		Ethnic Food Stores (EF)		Fast Food Restaurants (FF)		Supermarkert/Grocery Stores (SG)		Ethnic Food Stores (EF)		Fast Food Restaurants (FF)	
**Psychosocial Variables**							*b*					
	FS	**.09****	FS	**.09****	FS	**.09****	FS	-0.003	FS	0.0003	FS	-0.005
Food Security (FS)												
(n = 480/420)	E	**-0.06***	E	**-.06***	E	**-.06***	E	-0.03	E	-0.03	E	-0.03
Education (E)	SG	0.05	EF	-0.09	FF	0.07	SG	**.14***	EF	-0.01	FF	**.13***
Fruit/Vegetable Affordable (FA) (n = 481/449)	FA	**.12***	FA	**.12***	FA	**.12***	FA	0.005	FA	-0.003	FA	-0.00001
	E	**-.08****	E	**-.08***	E	**-.08****	E	-0.03	E	-0.03	E	-0.03
	SG	0.07	EF	-0.070	FF	0.09	SG	**.13***	EF	-0.02	FF	**.12***
Food Management (FM)												
(n = 476/409)	FM	**-.006***	FM	**-.006***	FM	**-.006***	FM	-0.002	FM	-0.002	FM	-0.002
	E	**-0.10****	E	**-.09****	E	**-.10****	E	-0.03	E	-0.03	E	-0.03
	SG	0.02	EF	-0.08	FF	0.12	SG	**.14***	EF	0.04	FF	**.16***
Food Unaffordable (FU)												
(n = 476/435)	FU	**-.14***	FU	**-.15***	FU	**-.14***	FU	0.01	FU	0.02	FU	0.03
	E	**-.07***	E	**-.06***	E	**-.07***	E	-0.02	E	-0.02	E	-0.02
	SG	0.07	EF	-0.07	FF	0.08	SG	**.12***	EF	-0.04	FF	**.13***
Grocery/Exercise Access (GE) (n = 481/443)	GE	0.08	GE	0.06	GE	0.07	GE	-0.002	GE	-0.02	GE	**-0.01**
	E	**-.09****	E	**-.09****	E	**-.09****	E	-0.03	E	-0.03	E	-0.03
	SG	0.11	EF	-0.06	FF	0.1	SG	**.13***	EF	-0.0005	FF	**.13***
Weight Norms (WN)												
(n = 481/432)	WN	**.10****	WN	**.09****	WN	**.09****	WN	0.03	WN	0.03	WN	0.03
	E	**-0.07***	E	**-.07***	E	**-.08***	E	-0.04	E	-0.02	E	-0.03
	SG	0.11	EF	-0.05	FF	**0.11***	SG	**.12***	EF	0.004	FF	**.11***
Eating Norms (EN) (n =												■
480/485)	EN	**-.13****	EN	**-.12****	EN	**-.13****	EN	**-.08****	EN	**-.08****	EN	■
	E	**-0.05***	E	**-.05***	E	**-.05***	E	-0.01	E	-0.01	E	■
	SG	0.1	EF	-0.05	FF	0.12	SG	**.14***	EF	-0.007	FF	■

* all models adjusted for age; Hispanic models adjusted for acculturation; ** p ≤ .001; *p ≤ .05; ■ significant interaction terms in main analyses and were not included in these supplemental analyses.

Supplemental Table 2c. For models without significant interaction terms: Associations of psychosocial and environmental variables **with percent calories from fat**, by ethnicity, adjusted for age.

	Caucasian (n=493)						Hispanic (n=509)					
	**Area-Level Variables**											
	Supermarkert/Grocery Stores (SG)		Ethnic Food Stores (EF)		Fast Food Restaurants (FF)		Supermarkert/Grocery Stores (SG)		Ethnic Food Stores (EF)		Fast Food Restaurants (FF)	
**Psychosocial Variables**	*b*											
Food Security (FS) (n = 480/420) Education (E)	FS	-0.23	FS	-0.34	FS	-0.32	FS		FS		FS	
	E	-0.3	E	-0.38	E	-0.33	E		E		E	
	SG	-1.65	EF	1.65	FF	1.72	SG		EF		FF	
Fruit/Vegetable Affordable (FA) (n = 481/449)	FA	0.62	FA	0.580	FA	0.59	FA	0.13	FA	0.2	FA	0.22
	E	-0.12	E	-0.160	E	-0.13	E	0.39	E	0.38	E	0.38
	SG	-1.42	EF	1.350	FF	1.78	SG	-1.34	EF	0.03	FF	-0.92
Food Management (FM) (n = 476/409)	FM	-0.01	FM	-0.01	FM	-0.008	FM	-0.03	FM	-0.03	FM	-0.03
	E	-0.04	E	-0.09	E	-0.05	E	0.5	E	0.49	E	0.48
	SG	-1.09	EF	**2.21***	FF	1.94	SG	-1.76	EF	0.29	FF	1.12
Food Unaffordable (FU) (n = 476/435)	FU	-0.36	FU	-0.24	FU	-0.29	FU	-0.43	FU	-0.48	FU	-0.49
	E	-0.17	E	-0.23	E	-0.19	E	-0.33	E	0.32	E	0.33
	SG	-1.6	EF	1.36	FF	1.74	SG	-0.96	EF	0.4	FF	-0.41
Grocery/Exercise Access (GE) (n = 481/443)	GE	-0.9	GE	-0.68	GE	-0.6	GE	0.25	GE	0.47	GE	0.41
	E	-0.25	E	-0.29	E	-0.24	E	0.35	E	0.33	E	0.33
	SG	-2.06	EF	1.64	FF	1.75	SG	-1.23	EF	0.05	FF	-1.04
Weight Norms (WN) (n = 481/432)	WN	-0.16	WN	-0.08	WN	-0.11	WN	0.04	WN	0.05	WN	0.05
	E	-0.26	E	-0.29	E	-0.25	E	0.43	E	0.41	E	0.41
	SG	2.00	EF	1.58	FF	1.75	SG	-1.56	EF	-0.19	FF	-1.08
Eating Norms (EN) (n = 480/485)	EN	-0.13	EN	-0.15	EN	-0.15	EN	**-1.12****	EN	**-1.15****	EN	**-1.21****
	E	-0.15	E	-0.19	E	-0.14	E	**0.67***	E	**.67***	E	**.67***
	SG	-1.92	EF	1.64	FF	1.83	SG	-1.42	EF	-0.15	FF	1.71

* all models adjusted for age; Hispanic models adjusted for acculturation; ** p ≤ .001; *p ≤ .05; x = no significant relationship identified in full models; ■ = significant interaction terms in main analyses and were not included in these supplemental analyses.

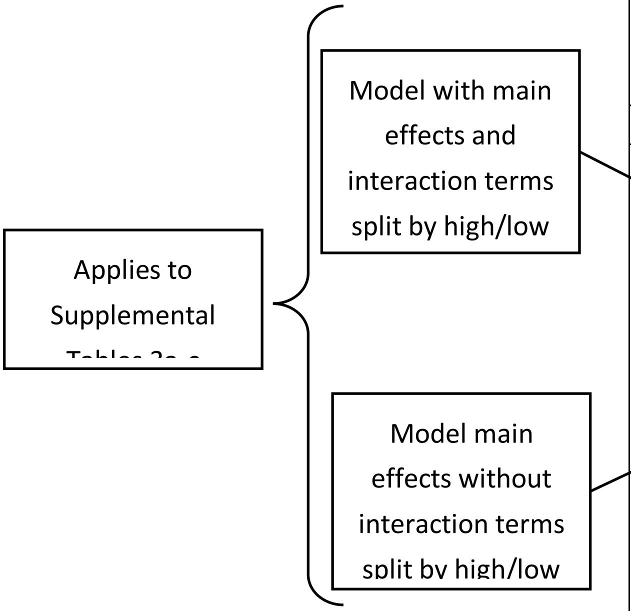

Supplemental Table 3a. For models with significant interactions in main analysis: associations of psychosocial and environmental variables **with fruit and vegetable consumption**, by ethnicity, split by education, adjusted for age and acculturation.

	Hispanic (n=509)			
	Education High (>=High School) (n = 123)		Education Low (<= Some High School) (n = 461)	
**Psychosocial Variables**	**Area-Level Variables**			
	Ethnic Food Stores (EF)			
	b			
Grocery/Exercise Access (GE) (n = 481/443)	GE	-0.23	GE	-0.26
	E	-0.07	E	0.006
	EF	0.24	EF	0.54
	GE x E	0.06	GE x E	0.03
	EF x E	-0.04	EF x E	-0.12
Grocery/Exercise Access (GE) (n = 481/443)	GE	-0.07	GE	-0.09
	E	0.02	E	0.005
	EF	0.12	EF	-0.12
Weight Norms (WN) (n = 481/432)	WN	0.08	WN	0.03
	E	0.08	E	0.05
	EF	0.58	EF	0.18
	WN x E	-0.02	WN x E	-0.02
	EF x E	-0.12	EF x E	-0.01
Weight Norms (WN) (n = 481/432)	WN	-0.01	WN	-0.03
	E	-0.02	E	-0.005
	EF	-0.11	EF	**.15***
Eating Norms (EN) (n = 480/485)	EN	**.61***	EN	0.05
	E	0.35	E	-0.02
	EF	0.75	EF	0.26
	EN x E	-0.11	EN x E	0.02
	EF x E	-0.16	EF x E	-0.04
Eating Norms (EN) (n = 480/485)	EN	0.03	EN	**.11****
	E	-0.006	E	-0.009
	EF	-0.12	EF	**.15***

* all models adjusted for age; Hispanic models adjusted for acculturation; ** $p \leq .001$; *$p \leq .05$;

Supplemental Table 3b. For models that had significant interactions in main analysis: Associations of psychosocial and environmental variables with **soft drink** consumption, by ethnicity, split by education, adjusted for age and acculturation.

	**Hispanic (n=509)**			
	Education High (>=High School) (n = 123)		Education Low (<= Some High School (n = 461)	
**Psychosocial Variables**	**Area-Level Variables**			
	Fast Food Restaurants (FF)			
	b			
Eating Norms (EN) (n = 480/485)	EN	-0.02	EN	-0.01
	E	-0.29	E	-0.03
	FF	-1.86	FF	-0.23
	EN x E	-0.02	EN x E	-0.02
	FF x E	0.35	FF x E	**-.12***
Eating Norms (EN) (n = 480/485)	EN	**-.14***	EN	**-0.05***
	E	-0.18	E	0.01
	FF	0.03	FF	0.09

* all models adjusted for age; Hispanic models adjusted for acculturation; ** p ≤ .001; *p ≤ .05;

Supplemental Table 3c. For models that had significant interactions in main analysis: Associations of psychosocial and environmental variables with **percent calories from fat**, by ethnicity, split by education, adjusted for age and acculturation.

	Hispanic (n=509)											
	Education High (>=High School)						Education Low (<= Some High School)					
**Psychosocial Variables**	**Area-Level Variables**											
	Supermarkert/Groc		Ethnic Food Stores		Fast Food		Supermarkert/Groc		Ethnic Food Stores		Fast Food	
	b						*b*					
Food Security (FS) (n= 411/333)	FS	-3.33	FS	-1.28	FS	-2.78	FS	**-1.78***	FS	**-1.77***	FS	**-1.79***
Education E	E	-0.74	E	-2.71	E	0.78	E	-0.36	E	-0.25	E	-0.37
	SG	-3.48	EF	-28.68	FF	12.7	SG	-0.44	EF	**2.09**	FF	-2.23
	FS x E	0.67	FS x E	0.3	FS x E	0.58	FS x E	0.55	FS x E	0.54	FS x E	0.55
	SG x E	0.05	EF x E	4.83	FF x E	-2.47	SG x E	-0.11	EF x E	-0.36	FF x E	0.49
Food Security (FS) (n = 480/420)	FS	0.36	FS	0.4	FS	0.41	FS	-0.25	FS	-0.29	FS	-0.25
Education (E)	E	0.34	E	0.18	E	0.5	E	0.6	E	0.52	E	0.61
	SG	-2.92	EF	-2.3	FF	-0.78	SG	-0.74	EF	1.2	FF	-0.83

* all models adjusted for age; Hispanic models adjusted for acculturation; ** p ≤ .001; *p ≤ .05;
